# Associations of Night-Shift Work, Sleep Duration, and Physical Activity with Burnout Among Healthcare Workers in Bucharest, Romania: A Cross-Sectional Study Using the Burnout Assessment Tool

**DOI:** 10.3390/healthcare14162513

**Published:** 2026-08-12

**Authors:** Nadia El-Agha, Adina-Diana Moldovan, Sorin-Ioan Tudorache, Cristina Spirea, Roxana Nemeş

**Affiliations:** 1 Medical Doctoral School, Titu Maiorescu University, 040317 Bucharest, Romania; nadia.el-agha@prof.utm.ro; 2 Department of Medico-Surgical Disciplines, Faculty of Medicine, Titu Maiorescu University, 031593 Bucharest, Romania; 3 Department for the Assessment of Risk Factors in the Living and Working Environment, Occupational Medicine and Occupational Diseases Unit, Public Health Directorate of Bucharest Municipality, 021578 Bucharest, Romania; 4 Department of Preclinical Disciplines, Faculty of Medicine, Titu Maiorescu University, 031593 Bucharest, Romania; roxana.nemes@prof.utm.ro; 5 Department of Work and Organizational Psychology, Sanador Clinical Hospital, 010991 Bucharest, Romania; cristinaspirea@gmail.com

**Keywords:** burnout, healthcare workers, night-shift work, sleep duration, physical activity, occupational stress, occupational health

## Abstract

**Highlights:**

**What are the main findings?**

- Night-shift workers had substantially higher BAT total and dimensional burnout scores than workers without night shifts, and night-shift work showed the strongest adjusted association with burnout.
- Shorter sleep duration remained independently associated with higher burnout, whereas physical activity was associated with lower burnout only in unadjusted analyses.

**What are the implications of the main findings?**

- Occupational health programs should prioritize work-schedule organization, recovery opportunities, and sleep-health support for healthcare workers.
- Longitudinal studies using detailed shift exposure and validated or objective sleep and physical-activity measures are needed before causal or intervention conclusions can be drawn.

**Abstract:**

**Background/Objectives:** Occupational burnout is a major challenge among healthcare workers. Night-shift work, insufficient sleep, and lifestyle factors may contribute to higher burnout levels. This study examined associations of night-shift work, sleep duration, and physical activity with burnout using the Burnout Assessment Tool (BAT). **Methods:** This cross-sectional study included 213 healthcare workers undergoing routine occupational health assessments in Bucharest, Romania, between January and April 2026. Burnout was assessed using the BAT-23 core questionnaire and BAT Secondary Symptoms module. Data on night-shift work, sleep duration, physical activity, age, sex, and professional experience were analyzed using group comparisons, correlations, and multivariable linear regression. **Results:** Night-shift workers had higher burnout scores than non-night-shift workers (3.36 ± 0.43 vs. 2.44 ± 0.41, *p* < 0.001), while physically active participants had lower scores than sedentary workers (2.46 ± 0.44 vs. 3.23 ± 0.52, *p* < 0.001). Burnout correlated negatively with sleep duration (r = −0.336, *p* < 0.001) and physical activity (r = −0.655, *p* < 0.001), and positively with night-shift work (r = 0.798, *p* < 0.001). In adjusted analysis, night-shift work (B = 0.831, 95% CI 0.637–1.024, *p* < 0.001), shorter sleep duration (B = −0.079, 95% CI −0.127 to −0.031, *p* = 0.001), and female sex (B = 0.162, 95% CI 0.029–0.295, *p* = 0.017; male = 0, female = 1) were associated with higher burnout; the model explained 58.6% of the variance. **Conclusions:** Night-shift work and shorter self-reported sleep duration were independently associated with higher burnout. Physical activity was associated with lower burnout only in unadjusted analyses. Owing to the cross-sectional design and self-reported exposures, findings should be interpreted as associations and confirmed longitudinally.

## 1. Introduction

Occupational burnout has become an increasingly important challenge for healthcare systems worldwide and is currently recognized by the World Health Organization as an occupational phenomenon resulting from chronic workplace stress that has not been successfully managed [1]. Healthcare professionals are routinely exposed to high workloads, emotional demands, time pressure, and substantial clinical responsibility [2,3]. High burnout prevalence has been documented among physicians [4] and nurses [5]. The COVID-19 period introduced additional workplace mental-health pressures, which should be distinguished from the broader evidence on burnout determinants [6].

Burnout has important consequences not only for healthcare workers but also for healthcare organizations and patients. Previous studies have associated burnout with reduced job satisfaction, absenteeism, decreased work performance, increased turnover intentions, medical errors, and lower quality of patient care [2,3,4]. Consequently, identifying modifiable occupational and lifestyle factors associated with burnout has become an important objective in occupational health research.

Among healthcare workers, night-shift work represents one of the most common occupational exposures associated with psychological strain. Modern healthcare systems require continuous patient care, making shift work and overnight duties unavoidable in many medical professions. Although essential for healthcare delivery, night-shift work may disrupt circadian rhythms, impair recovery processes, and negatively affect both physical and mental health [7,8,9]. Previous research has shown that healthcare professionals working night shifts experience higher levels of fatigue, emotional exhaustion, sleep disturbances, and occupational stress compared with daytime workers [7,8,9,10].

Sleep is increasingly recognized as a key determinant of psychological well-being and occupational functioning. Adequate sleep duration is essential for cognitive performance, emotional regulation, stress resilience, and recovery from work-related demands. However, healthcare workers frequently report insufficient sleep due to shift schedules, extended working hours, on-call duties, and increasing clinical workloads [10,11]. Sleep deprivation has been associated with impaired attention, reduced decision-making capacity, emotional dysregulation, and a greater risk of burnout [10,11,12]. Given the demanding nature of healthcare work, understanding the relationship between sleep duration and burnout may have important implications for occupational health interventions.

Lifestyle factors may also influence the development and progression of burnout. In particular, regular physical activity has been associated with numerous psychological and physiological benefits, including improved mood, reduced stress, better sleep quality, and enhanced overall well-being [12,13]. Several studies have suggested that physically active individuals may experience lower levels of occupational stress and burnout [12,13]. Nevertheless, the extent to which physical activity independently contributes to burnout risk among healthcare workers remains incompletely understood, particularly when occupational factors such as night-shift work and sleep duration are considered simultaneously.

The assessment of burnout has evolved considerably in recent years. Although the Maslach Burnout Inventory has traditionally been the most widely used instrument, the Burnout Assessment Tool (BAT) was developed to provide a broader and more comprehensive evaluation of burnout [14,15]. The BAT conceptualizes burnout as a multidimensional syndrome encompassing four core dimensions—exhaustion, mental distance, cognitive impairment, and emotional impairment—as well as secondary symptoms including psychological distress and psychosomatic complaints [14,15]. This multidimensional approach may provide a more detailed understanding of burnout manifestations among healthcare professionals.

Romania provides a relevant context for occupational-health research among healthcare workers. Recent international assessments have identified persistent workforce-retention challenges, demanding working conditions, high workloads, and night-shift burden within the Romanian health system. These pressures make the study of burnout and recovery particularly relevant, while the concentration of the present sample in Bucharest requires caution when extrapolating to other regions and rural healthcare settings [16,17,18].

Night-shift work, insufficient sleep, and physical inactivity have each been associated with burnout, but these exposures are also behaviorally and occupationally interrelated. Much of the existing literature evaluates one factor at a time or uses burnout instruments that do not capture the full BAT symptom framework. Consequently, it remains unclear whether the associations of sleep duration and physical activity persist when current night-shift work and demographic factors are considered simultaneously. Evidence from occupational-health samples in Eastern Europe, including Romania, is also limited, although Romanian-language psychometric evidence is available for a short BAT version.

The present study does not seek to estimate national burnout prevalence. Its contribution is to quantify the conditional associations of three potentially modifiable, interrelated factors with BAT-measured burnout in a heterogeneous occupational-health sample from Bucharest and to identify measurement and design priorities for larger longitudinal studies.

Accordingly, we examined the associations of current night-shift work, self-reported sleep duration, and self-reported physical activity with BAT total and dimension scores among healthcare workers undergoing occupational health assessments in Bucharest. We hypothesized that night-shift work and shorter sleep duration would be associated with higher burnout, while physical activity would be associated with lower burnout before and after mutual adjustment.

## 2. Materials and Methods

### 2.1. Study Design and Participants

This cross-sectional study was conducted between January and April 2026 and aimed to examine the associations of occupational and lifestyle-related factors with burnout among healthcare workers.

This was a non-probability, consecutive sample recruited through occupational medicine services in Bucharest, Romania. The study was designed to evaluate associations within the recruited occupational-health cohort and was not intended to provide a nationally representative estimate of burnout among all Romanian healthcare workers. A total of 213 healthcare workers met the eligibility criteria and were included in the final analysis. The sample comprised all eligible participants recruited consecutively during the predefined study period. No formal a priori power calculation was used to set recruitment. A sensitivity analysis for a fixed-effects multiple linear regression with six predictors, *n* = 213, α = 0.05, and 80% power indicated a minimum detectable overall effect size of f^2^ = 0.0659, equivalent to R^2^ ≈ 0.062. Regression coefficients are reported with 95% confidence intervals. Diagnostics showed no evidence of heteroscedasticity (Breusch–Pagan *p* = 0.163; White *p* = 0.908) and no severe multicollinearity (VIF range 1.048–3.094), while residual normality was not fully met (Shapiro–Wilk W = 0.974, *p* < 0.001). Influence checks showed maximum Cook’s distance = 0.064 The total number of eligible workers approached was not preserved in contemporaneous recruitment records; consequently, a response rate could not be calculated. This limits assessment of non-response and selection bias.

The study population comprised physicians, resident physicians, nurses, nursing assistants, orderlies, stretcher-bearers, physiotherapists, laboratory personnel, medical registrars, secretarial staff, and other healthcare professionals directly involved in patient care and hospital activities.

Administrative personnel were excluded from the present analysis to obtain a more homogeneous cohort and to focus on occupational exposures specific to healthcare professions, particularly night-shift work, sleep disruption, and work-related stress.

Inclusion criteria were age 18 years or older, active employment in a healthcare profession, completion of the BAT-23, and provision of written informed consent. Exclusion criteria were incomplete questionnaire data, missing information for the occupational variables of interest, refusal to participate, or withdrawal of consent.

### 2.2. Ethical Considerations

The study was conducted in accordance with the ethical principles outlined in the Declaration of Helsinki. Ethical approval was obtained from the Ethics Committee of the clinical hospital where the study was conducted (Bagdasar-Arseni Emergency Clinical Hospital, Bucharest, Romania—protocol approval No. 2224/20 January 2026); also, this study was approved by the Ethics Committee of Titu Maiorescu University (protocol code 20/13 July 2026). All participants received detailed information regarding the purpose and procedures of the study and provided written informed consent prior to enrollment.

### 2.3. Data Collection

Data were collected using a structured questionnaire and occupational health records The following demographic and occupational variables were recorded: age (years), sex, professional experience (years), occupational category, night-shift work, sleep duration (hours per night), physical activity status. Night-shift work was coded as present (1) or absent (0) according to participants’ current work schedule. Sleep duration was assessed through self-report and recorded as the average number of hours slept per night. Physical activity status was assessed through self-report. Participants reporting regular leisure-time physical activity were classified as physically active (1), whereas those reporting no regular physical activity were classified as sedentary (0). Sex was coded as female (0) and male (1).

Occupational burnout was assessed using the Burnout Assessment Tool (BAT-23), a validated instrument developed by Schaufeli and colleagues for the multidimensional assessment of burnout [14,15].

Participants completed the official 23-item core Burnout Assessment Tool (BAT-23) for Romania (https://burnoutassessmenttool.be/wp-content/uploads/2020/08/BAT-Romanian.pdf, accessed on 5 June 2026) together with the 10-item BAT Secondary Symptoms module. The BAT-23 core comprises exhaustion (8 items), mental distance (5 items), cognitive impairment (5 items), and emotional impairment (5 items). Items are rated from 1 (“never”) to 5 (“always”). Each dimension score was calculated as the arithmetic mean of its constituent items, and the BAT core total was calculated as the arithmetic mean of all 23 core items; therefore, all core scores ranged from 1 to 5, with higher scores indicating more frequent burnout symptoms. The Secondary Symptoms score was calculated separately as the mean of the 10 secondary-symptom items and was not included in the BAT-23 core total. No standardization or transformation was applied. Psychometric evidence has been reported for a Romanian short version of the BAT, although this does not constitute country-specific validation of clinical cut-offs for the full BAT-23.

BAT scores were analyzed continuously. Published pooled European cut-offs were not used to estimate the prevalence of at-risk or severe burnout because Romanian-specific clinical cut-offs for the full BAT-23 have not been established; applying external thresholds could therefore lead to misclassification.

### 2.4. Statistical Analysis

Statistical analyses were performed using IBM SPSS Statistics version 20.0 (IBM Corp., Armonk, NY, USA). Continuous variables were summarized using mean ± standard deviation and, for non-parametric group comparisons, median and interquartile range (IQR). Distributional form was assessed using histograms, Q-Q plots, and the Shapiro–Wilk test, with attention to outliers and variance differences. Because BAT total scores showed non-normal overall and group-specific distributions on Shapiro–Wilk testing (overall W = 0.983, *p* = 0.010; all main BAT group strata *p* ≤ 0.001) and visible tail observations, between-group comparisons of BAT scores were performed using the Mann–Whitney U test. Variance homogeneity was not rejected for the main BAT total contrasts (Levene *p* = 0.715 for night-shift groups and *p* = 0.239 for physical-activity groups). The U statistic, two-sided *p* value, rank-biserial correlation, and r = |Z|/√N were reported. Associations between burnout scores and demographic or occupational variables were evaluated using Spearman’s rank correlation coefficient. Continuous-variable distributions were examined using histograms, Q-Q plots, and the Shapiro–Wilk test, with attention to outliers and variance differences. Because BAT total scores showed non-normal overall and group-specific distributions on Shapiro–Wilk testing (overall W = 0.983, *p* = 0.010; all main BAT group strata *p* ≤ 0.001) and visible tail observations, BAT group comparisons were performed using the Mann–Whitney U test and are reported as median (IQR), together with U, p, rank-biserial effect size, and r = |Z|/√N; mean ± SD is retained as a supplementary descriptive measure. Regression assumptions were evaluated using residual and influence diagnostics, and multicollinearity was assessed using tolerance and VIF.

The following variables were entered into the regression model: sleep duration, night-shift work, physical activity, age, sex, professional experience. Spearman rank correlations were also calculated among night-shift work, sleep duration, physical activity, and BAT total score to describe the interrelationships among the principal study variables.

Unstandardized regression coefficients (B), 95% confidence intervals (95% CI), and *p* values were reported. Model performance was evaluated using the coefficient of determination (R^2^), adjusted R^2^, and the F statistic. All statistical tests were two-tailed and a *p* value <0.05 was considered statistically significant.

Before interpreting the multivariable model, linearity was evaluated using partial-residual and predictor-versus-residual plots, residual normality using a histogram and Q-Q plot, and homoscedasticity using residual-versus-fitted plots. Multicollinearity was assessed using tolerance and variance inflation factors (VIFs), and influential observations were examined using standardized residuals, leverage values, and Cook’s distance. Both unstandardized coefficients (B) and standardized coefficients (β) were reported.

## 3. Results

### 3.1. Participant Characteristics

A total of 213 healthcare workers were included in the study. The study population included several healthcare occupational categories. Nurses represented the largest professional group (51.6%), followed by nursing assistants (23.5%), orderlies/stretcher-bearers (9.4%), physicians and resident physicians (8.5%), and medical registrars or secretarial staff (4.7%). The complete distribution of occupational categories is presented in Table 1.

The mean age of the participants was 47.09 ± 9.24 years, and the majority were women (164/213, 77.0%). The mean professional experience was 12.69 ± 7.75 years.

Night-shift work was reported by 116 participants (54.5%), while 97 (45.5%) did not perform night shifts. Regular physical activity was reported by 80 participants (37.6%), whereas 133 (62.4%) were classified as sedentary. Physical-activity status differed markedly between shift groups: among workers without night shifts, 78/97 (80.4%) were physically active and 19/97 (19.6%) were sedentary; among night-shift workers, 2/116 (1.7%) were physically active and 114/116 (98.3%) were sedentary (χ^2^(1) = 139.47, *p* < 0.001).

The mean self-reported sleep duration was 5.13 ± 1.20 h per night. The mean BAT total score for the study population was 2.94 ± 0.62. Participant characteristics are summarized in Table 2.

### 3.2. Burnout According to Night Shift Work

**Figure 1:**
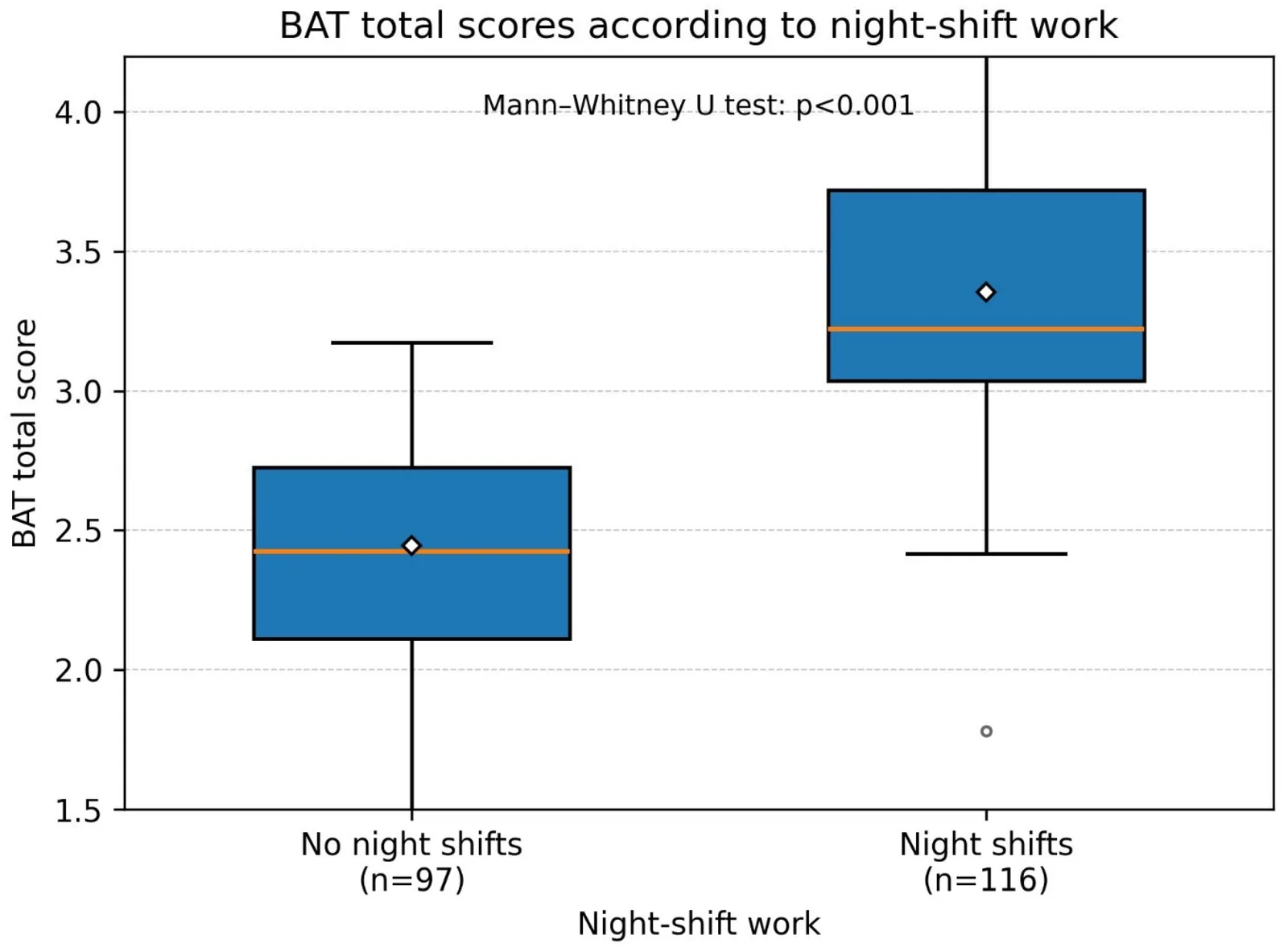
Distribution of BAT total scores according to night-shift work among healthcare workers. Healthcare workers performing night shifts exhibited significantly higher burnout scores compared with those not performing night shifts (*p* < 0.001). This observation had standardized residual = −4.12, leverage = 0.018, and Cook’s distance = 0.045. A sensitivity analysis excluding this observation did not materially change the main findings: night-shift work remained strongly associated with burnout (B = 0.845, 95% CI 0.659–1.030, *p* < 0.001), sleep duration remained independently associated with burnout (B = −0.084, 95% CI −0.129 to −0.038, *p* < 0.001), and physical activity remained non-significant (B = −0.043, *p* = 0.645). Excluding all eight observations with Cook’s distance > 4/*n* also did not change the conclusions (night-shift work: B = 0.823, 95% CI 0.652–0.993, *p* < 0.001). The horizontal line within each box represents the median, the white diamond indicates the arithmetic mean, the box represents the interquartile range, the whiskers indicate the most extreme non-outlying observations, and the hollow circle denotes an outlier located more than 1.5 interquartile ranges beyond the first or third quartile.

Healthcare workers performing night shifts exhibited significantly higher burnout scores than those without night shifts (3.36 ± 0.43 vs. 2.44 ± 0.41, *p* < 0.001). Detailed comparisons of BAT scores according to night-shift work are presented in Table 3 while the distribution of BAT total scores is illustrated in Figure 1.

The standardized mean difference in BAT total score between night-shift and non-night-shift workers was very large (Cohen’s d = 2.16, 95% CI 1.82–2.50). The non-parametric comparison was also large: Mann–Whitney U = 422.0, *p* < 0.001, rank-biserial r = 0.925 (bootstrap 95% CI 0.860–0.976), and r = |Z|/√N = 0.796.

Among BAT dimensions, night-shift workers reported significantly higher exhaustion scores (3.45 ± 0.77 vs. 2.60 ± 0.70, *p* < 0.001), mental distance scores (2.81 ± 0.58 vs. 2.45 ± 0.61, *p* < 0.001), cognitive impairment scores (2.72 ± 0.50 vs. 2.36 ± 0.59, *p* < 0.001), emotional impairment scores (2.76 ± 0.47 vs. 2.45 ± 0.59, *p* < 0.001), and secondary symptom scores (3.06 ± 0.63 vs. 2.85 ± 0.71, *p* = 0.018) (Table 3).

### 3.3. Burnout According to Physical Activity

Physically active healthcare workers demonstrated significantly lower burnout scores than sedentary participants (2.46 ± 0.44 vs. 3.23 ± 0.52, *p* < 0.001).

Significant differences were observed for exhaustion (2.66 ± 0.72 vs. 3.31 ± 0.83, *p* < 0.001), mental distance (2.50 ± 0.63 vs. 2.74 ± 0.60, *p* = 0.007), cognitive impairment (2.41 ± 0.62 vs. 2.65 ± 0.53, *p* = 0.001), and emotional impairment (2.53 ± 0.60 vs. 2.68 ± 0.51, *p* = 0.043). Although secondary symptom scores were lower among physically active participants (2.91 ± 0.74 vs. 3.00 ± 0.63), the difference did not reach statistical significance (*p* = 0.289). Comparisons of BAT scores according to physical activity are presented in Table 4.

The absolute standardized mean difference in BAT total score between sedentary and physically active participants was also large (Cohen’s d = 1.57, 95% CI 1.26–1.89). The non-parametric comparison confirmed a large effect in the same direction: Mann–Whitney U = 9471.0 for sedentary versus physically active participants, *p* < 0.001, rank-biserial r = 0.780 (bootstrap 95% CI 0.682–0.868), and r = |Z|/√N = 0.653.

### 3.4. Associations Between Burnout and Occupational Factors

Burnout showed significant associations with occupational and lifestyle factors. Higher burnout scores were observed among workers performing night shifts, whereas longer sleep duration and regular physical activity were associated with lower burnout levels.

The principal exposures were interrelated: night-shift work correlated with sleep duration (ρ = −0.245, *p* < 0.001) and physical activity (ρ = −0.809, *p* < 0.001), while sleep duration correlated with physical activity (ρ = 0.185, *p* = 0.007). These relationships were considered when interpreting the mutually adjusted regression coefficients.

No significant associations were identified between burnout and age, sex, or professional experience in univariate analyses. Spearman correlation coefficients are presented in Table 5.

### 3.5. Factors Independently Associated with Burnout

A multivariable linear regression analysis was performed to identify factors independently associated with burnout.

Regression diagnostics showed acceptable functional form by Ramsey RESET (F = 0.620, *p* = 0.432) and no evidence of heteroscedasticity by Breusch–Pagan testing (*p* = 0.163) or White testing (*p* = 0.908). Residual normality was not fully met (Shapiro–Wilk W = 0.974, *p* < 0.001; Jarque–Bera *p* < 0.001), mainly because of tail observations, and this should be acknowledged. Tolerance values ranged from 0.323 to 0.954 and VIF values from 1.048 to 3.094, indicating no severe multicollinearity by conventional thresholds, although night-shift work and physical activity shared substantial variance. The largest Cook’s distance was 0.064; 8 observations exceeded 4/*n* and 2 observations had standardized residuals exceeding |3|. Sensitivity analyses excluding the lowest night-shift BAT observation and excluding the observations with Cook’s distance > 4/*n* did not materially change the main conclusions (Table 6).

Night-shift work remained the factor most strongly associated with higher burnout scores (B = 0.831, *p* < 0.001). Shorter sleep duration was also independently associated with higher burnout levels (B = −0.079, *p* = 0.001).

Although physical activity was associated with lower burnout scores in univariate analyses, it did not remain significantly associated with burnout after adjustment for occupational and demographic variables (*p* = 0.655).

Female sex was independently associated with higher burnout scores (B = −0.162, *p* = 0.017). As sex was coded as 0 = female and 1 = male, the negative coefficient indicates higher burnout scores among women. Professional experience was not significantly associated with burnout in the adjusted model (*p* = 0.074).

The overall regression model explained 58.6% of the variance in burnout scores (R^2^ = 0.586; adjusted R^2^ = 0.574; F = 48.52; *p* < 0.001). Results of the multivariable regression analysis are presented in Table 7.

## 4. Discussion

The present study found that night-shift work and shorter sleep duration were independently associated with higher BAT total scores. Night-shift workers also reported higher scores across all BAT dimensions, whereas physical activity was associated with lower burnout only in unadjusted analyses. The magnitude of the observed associations was larger than is commonly encountered in observational burnout research. This pronounced separation may partly reflect the composition of the occupational-health sample, the binary exposure classifications, and correlations among work schedule, sleep, and physical activity. In addition, selection bias, residual confounding, and common-method variance from contemporaneous self-report may have inflated or otherwise distorted the estimates. Accordingly, effect sizes should be interpreted cautiously and require replication in independent, prospectively sampled cohorts.

Among the examined factors, night-shift work showed the strongest adjusted association with burnout. This finding is consistent with literature linking night work and irregular schedules with fatigue, sleep disturbance, emotional exhaustion, and occupational stress among healthcare professionals [7,8,9,10]. The present cross-sectional data do not identify the mechanism underlying this association. Similar findings have been reported in both European and North American healthcare settings, where night-shift work has consistently been associated with higher burnout levels among physicians, nurses, and other healthcare professionals [2,6,7,8,9,10].

The observed association between night-shift work and exhaustion is particularly relevant because exhaustion is widely considered the core component of burnout [14,15,19]. Repeated exposure to occupational demands without adequate recovery may progressively impair psychological resilience and contribute to chronic occupational stress. The significantly higher exhaustion scores observed among night-shift workers in the present study are consistent with this interpretation.

Sleep duration has also been found consistent with burnout. Participants reported a mean sleep duration of only 5.13 h per night, substantially below the duration generally recommended for healthy adults. Although sleep duration was self-reported, the findings are consistent with existing evidence indicating that insufficient sleep contributes to impaired recovery, emotional dysregulation, reduced cognitive performance, and increased vulnerability to burnout [10,11,12]. The present findings are also in agreement with international studies demonstrating that reduced sleep duration is associated with higher burnout levels among healthcare professionals [10,11,12]. Several European and American investigations have identified sleep deprivation as a significant contributor to occupational stress, emotional exhaustion, and decreased psychological well-being in healthcare settings [10,11,12].

The relatively short sleep duration observed in our cohort may reflect the demanding work schedules frequently encountered in healthcare settings. Many healthcare professionals are required to perform repeated night shifts and extended working hours while maintaining a high level of clinical responsibility. In addition, healthcare systems in many countries continue to face workforce shortages, increasing patient loads, and difficulties in recruiting additional staff. Such circumstances may contribute to more frequent night-shift schedules, reduced recovery time between shifts, and chronic sleep restriction among healthcare workers [2,7,8].

Physical activity was associated with lower burnout in unadjusted analyses but not after mutual adjustment for night-shift work, sleep duration, and demographic variables. This attenuation may reflect shared variance, confounding, or limited precision rather than evidence that physical activity is unimportant. Because the study is cross-sectional, it cannot establish temporal ordering or mediation among work schedule, sleep, physical activity, and burnout. Physically active participants demonstrated lower levels of exhaustion, mental distance, cognitive impairment, and emotional impairment. These findings support previous evidence suggesting that physical activity may represent an important protective factor against occupational stress [12,13]. Physical exercise has been associated with improvements in mood regulation, stress resilience, sleep quality, and overall psychological well-being [12,13]. Our findings are broadly consistent with previous international studies suggesting that physically active healthcare workers experience lower levels of burnout and psychological distress [12,13,14].

However, physical activity did not remain significantly associated with burnout in the multivariable regression model. This finding suggests that part of the observed relationship may be explained by confounding or shared variance with occupational factors, particularly sleep duration and night-shift work [12,13]. Nevertheless, the consistently lower burnout scores observed among physically active participants support the inclusion of health promotion strategies and workplace wellness programs as part of broader occupational health interventions.

Female sex was associated with higher burnout scores in the adjusted model. This secondary finding should be interpreted cautiously: women comprised 77.0% of the sample, sex was not associated with burnout in the unadjusted correlation analysis, and the model did not include occupation-specific workload, household or caregiving responsibilities, work-family conflict, or other psychosocial factors that may differ by sex. This finding is consistent with previous studies reporting increased burnout vulnerability among female healthcare professionals [2,3,4,5]. Several explanations have been proposed, including differences in occupational responsibilities, work–life balance challenges, emotional labor, caregiving obligations, and exposure to psychosocial stressors [2,3,4]. In healthcare settings, female professionals frequently face the combined demands of clinical work and family responsibilities, which may contribute to a greater risk of emotional exhaustion and burnout [2,3,4]. Further research is warranted to better understand the mechanisms underlying these sex-related differences and to identify targeted interventions aimed at reducing burnout risk among female healthcare workers.

The Romanian setting may shape the observed associations through local staffing patterns, work organization, and retention pressures; however, these contextual factors were not measured directly. The results should therefore be used to generate context-sensitive hypotheses and occupational-health priorities rather than to infer a uniquely Romanian effect.

The present study has several strengths. First, burnout was assessed using the Burnout Assessment Tool (BAT-23), a contemporary multidimensional instrument that provides a broader and more detailed evaluation of burnout compared with traditional burnout questionnaires [14,15,19]. By incorporating both core symptoms (exhaustion, mental distance, cognitive impairment, and emotional impairment) and secondary symptoms (psychological distress and psychosomatic complaints), the BAT allowed a more comprehensive assessment of occupational burnout and facilitated the exploration of specific symptom domains potentially affected by night-shift work, sleep duration, and physical activity [14,15].

Second, the study focused exclusively on healthcare workers, resulting in a relatively homogeneous population exposed to similar occupational stressors and work-related demands. The inclusion of multiple healthcare professions enhanced the applicability of the findings across different occupational groups within the healthcare sector. Third, several occupational and lifestyle factors, including night-shift work, sleep duration, and physical activity, were evaluated simultaneously, allowing a broader understanding of factors associated with burnout. Finally, the use of multivariable regression analysis enabled the identification of factors independently associated with burnout after adjustment for potential confounding variables.

Several limitations should be acknowledged. First, the cross-sectional design does not permit causal inferences regarding the observed associations. Consequently, it cannot be determined whether occupational factors contributed to burnout or whether burnout itself influenced participants’ lifestyle behaviors and sleep patterns. The sex distribution was imbalanced, and the adjusted sex coefficient may reflect residual confounding by occupation, work schedule, workload, caregiving, or other unmeasured social factors. The study was not designed or powered primarily to estimate sex-specific effects.

Second, sleep duration and physical activity were assessed through self-report, which may be subject to recall bias and reporting inaccuracies. Objective measures such as actigraphy or wearable activity monitoring devices were not available [10,11]. Because the principal exposures and the burnout outcome were recorded during the same occupational health encounter and were based largely on self-report, common-method variance and contemporaneous reporting bias may have inflated or otherwise distorted the observed associations. This possibility is particularly relevant to the large correlations involving physical activity, sleep duration, and burnout. The present design cannot quantify the extent of this bias, and the estimates should therefore be interpreted cautiously.

Third, sleep duration was evaluated as self-reported average sleep duration, whereas sleep quality, sleep fragmentation, and the presence of sleep disorders were not assessed. Therefore, the potential influence of these factors on burnout could not be evaluated.

Fourth, occupational exposure was classified according to the presence or absence of night-shift work, whereas the frequency, cumulative duration, and intensity of night-shift schedules were not assessed. Consequently, potential dose–response relationships between night-shift exposure and burnout could not be examined.

Fifth, the study population consisted of healthcare workers recruited during occupational health assessments and may not be fully representative of all healthcare professionals. Therefore, the generalizability of the findings should be interpreted with caution.

Sixth, the study did not evaluate additional psychosocial factors that may influence burnout, such as job satisfaction, workplace support, organizational climate, workload intensity, coping strategies, or work–life balance [2,3]. Future studies incorporating these variables may provide a more comprehensive understanding of the factors associated with occupational burnout among healthcare workers.

In addition, detailed information regarding the frequency and cumulative duration of night-shift work, weekly working hours, overtime, workload intensity, and validated measures of physical activity was not available. Residual confounding related to unmeasured occupational and psychosocial factors cannot therefore be excluded. Night-shift work and physical activity were classified using binary indicators, and sleep was represented by a single self-reported average. These measures do not capture shift frequency, consecutive night duties, cumulative night-work exposure, schedule rotation, sleep quality or fragmentation, or the frequency, intensity, and duration of physical activity. Exposure misclassification is therefore possible, and no dose–response relationship can be evaluated. Circadian disruption and impaired recovery are plausible mechanisms described in prior literature but were not measured or tested in this study.

Information regarding the number of healthcare workers invited to participate and the response rate was not systematically recorded, which may limit the assessment of potential selection bias.

Despite these limitations, the study provides valuable evidence regarding occupational factors associated with burnout among healthcare workers and highlights the potential importance of sleep duration and night-shift work as modifiable targets for occupational health interventions.

## 5. Conclusions

Night-shift work and shorter sleep duration were independently associated with higher burnout levels among healthcare workers. Although regular physical activity was associated with lower burnout scores in univariate analyses, this association was no longer statistically significant after adjustment for occupational and demographic factors. Future longitudinal studies incorporating detailed shift-work exposure, validated measures of sleep and physical activity, and additional psychosocial variables are needed to further clarify these associations.

These findings support closer attention to work schedules, recovery opportunities, and sleep health within occupational health surveillance; however, the cross-sectional design does not establish that modifying these factors will reduce burnout. Intervention effects should be evaluated prospectively. Because participants were recruited through collaborating occupational medicine services in Bucharest, the sample may differ from healthcare workers in other Romanian regions, rural settings, institutions, or occupational arrangements. The findings should therefore be interpreted as context-specific within-sample associations rather than national prevalence estimates.

## Figures and Tables

**Table 1:** Occupational categories of healthcare workers included in the study (*n* = 213).

Occupation Category	*n*	%
Nurses	110	51.6
Nursing assistants	50	23.5
Orderlies/Stretcher-bearers	20	9.4
Physicians and resident physicians	18	8.5
Medical registrars and secretarial staff	10	4.7
Laboratory personnel	4	1.9
Physiotherapists	1	0.5

Occupational categories were classified according to participants’ primary professional role within the healthcare institution.

**Table 2:** Baseline characteristics of healthcare workers included in the study (*n* = 213).

Variable	Overall (*n* = 213)	No Night Shifts (*n* = 97)	Night Shifts (*n* = 116)	*p* Value
Age, years	47.09 ± 9.24; 49.00 (40.00–53.00)	45.94 ± 9.73; 49.00 (38.00–53.00)	48.06 ± 8.72; 49.00 (44.00–53.25)	0.282
Female sex, *n* (%)	164 (77.0)	81 (83.5)	83 (71.6)	0.039
Professional experience, years	12.69 ± 7.75; 12.00 (7.00–15.00)	13.30 ± 9.92; 11.00 (5.00–20.00)	12.18 ± 5.29; 12.00 (10.00–14.00)	0.784
Physically active, *n* (%)	80 (37.6)	78 (80.4)	2 (1.7)	<0.001
Sedentary, *n* (%)	133 (62.4)	19 (19.6)	114 (98.3)	<0.001
Sleep duration, hours/night	5.13 ± 1.20; 5.00 (4.00–6.00)	5.48 ± 1.28; 5.00 (4.00–6.00)	4.84 ± 1.05; 4.50 (4.00–6.00)	<0.001
BAT total score	2.94 ± 0.62; 3.01 (2.44–3.25)	2.44 ± 0.41; 2.42 (2.11–2.73)	3.36 ± 0.43; 3.22 (3.04–3.72)	<0.001

**Table 3:** Burnout scores according to night shift work among healthcare workers.

Variable	No Night Shifts (*n* = 97)	Night Shifts (*n* = 116)	*p* Value
BAT total score	2.44 ± 0.41 2.42 (2.11–2.73)	3.36 ± 0.43 3.22 (3.04–3.72)	<0.001
Exhaustion	2.60 ± 0.70 2.63 (2.13–3.00)	3.45 ± 0.77 3.39 (3.00–3.91)	<0.001
Mental distance	2.45 ± 0.61 2.40 (2.00–2.80)	2.81 ± 0.58 2.80 (2.60–3.20)	<0.001
Cognitive impairment	2.36 ± 0.59 2.40 (2.00–2.80)	2.72 ± 0.50 2.80 (2.60–3.00)	<0.001
Emotional impairment	2.45 ± 0.59 2.40 (2.00–2.90)	2.76 ± 0.47 2.80 (2.40–3.00)	<0.001
Secondary symptoms	2.85 ± 0.71 2.77 (2.40–3.37)	3.06 ± 0.63 3.06 (2.60–3.52)	0.018

BAT = Burnout Assessment Tool; IQR = interquartile range; SD = standard deviation. Values are presented as mean ± SD and median (IQR). *p* values were calculated using two-sided Mann–Whitney U tests.

**Table 4:** Burnout scores according to physical activity among healthcare workers.

Variable	Sedentary (*n* = 133)	Physically Active (*n* = 80)	*p* Value
BAT Total Score	3.23 ± 0.52 3.18 (3.02–3.69)	2.46 ± 0.44 2.44 (2.11–2.73)	<0.001
Exhaustion	3.31 ± 0.83 3.30 (2.88–3.88)	2.66 ± 0.72 2.75 (2.18–3.13)	<0.001
Mental Distance	2.74 ± 0.60 2.70 (2.40–3.00)	2.50 ± 0.63 2.60 (2.20–3.00)	0.007
Cognitive Impairment	2.65 ± 0.53 2.60 (2.40–3.00)	2.41 ± 0.62 2.40 (2.00–2.80)	0.001
Emotional Impairment	2.68 ± 0.51 2.60 (2.40–3.00)	2.53 ± 0.60 2.40 (2.18–3.00)	0.043
Secondary Symptoms	3.00 ± 0.63 3.00 (2.60–3.43)	2.91 ± 0.74 2.83 (2.44–3.43)	0.289

BAT = Burnout Assessment Tool; IQR = interquartile range; SD = standard deviation. Values are presented as mean ± SD and median (IQR). *p* values were calculated using two-sided Mann–Whitney U tests.

**Table 5:** Correlations between burnout and occupational factors among healthcare workers (*n* = 213).

Variable	Spearman’s r	*p* Value
Sleep duration (hours/night)	−0.336	<0.001
Night shift work	0.798	<0.001
Physical activity	−0.655	<0.001
Age (years)	0.070	0.311
Sex	−0.001	0.992
Professional experience (years)	−0.021	0.761

BAT = Burnout Assessment Tool. Spearman correlation coefficients were calculated to assess associations between occupational factors and BAT total score.

**Table 6:** Regression diagnostics.

Predictor	Standardized β	Tolerance	VIF
Sleep duration	−0.153	0.910	1.099
Night-shift work	0.669	0.323	3.094
Physical activity	−0.034	0.343	2.914
Age	0.049	0.880	1.136
Female sex (vs. male)	0.110	0.954	1.048
Professional experience	−0.086	0.886	1.129

β = standardized regression coefficient; VIF = variance inflation factor.

**Table 7:** Multivariable linear regression analysis of factors associated with burnout among healthcare workers.

Variable	B Coefficient	95% CI	*p* Value
Sleep duration (hours/night)	−0.079	−0.127 to −0.031	0.001
Night shift work	0.831	0.637 to 1.024	<0.001
Physical activity	−0.044	−0.237 to 0.149	0.655
Age	0.003	−0.003 to 0.010	0.310
Sex	−0.162	−0.295 to −0.029	0.017
Professional experience	−0.007	−0.014 to 0.001	0.074

B = unstandardized regression coefficient. The regression model explained 58.6% of the variance in burnout scores (R^2^ = 0.586; adjusted R^2^ = 0.574; F = 48.52; *p* < 0.001).

## Data Availability

The data presented in this study are available on request from the corresponding author due to privacy restrictions enforced by the public clinical hospital where the study was conducted.

## Abbreviations

The following abbreviations are used in this manuscript:

BAT-23	23-item Burnout Assessment Tool
CI	Confidence interval
IQR	Interquartile range
SD	Standard deviation
VIF	Variance Inflation Factor
